# Postprandial Hyperglycaemia Screening and Pregnancy Outcomes‐Lessons From COVID ‐19

**DOI:** 10.1111/ajo.70014

**Published:** 2025-03-24

**Authors:** Beenu Bastian, Lisa Gaye Smithers, Ansar Kunjunju, Alexia Pape, Monique Francois

**Affiliations:** ^1^ School of Medical Indigenous and Health Sciences University of Wollongong Wollongong New South Wales Australia; ^2^ Illawarra Shoalhaven Diabetes Service Illawarra Shoalhaven Local Health District Wollongong New South Wales Australia; ^3^ School of Health and Society University of Wollongong Wollongong New South Wales Australia; ^4^ The Wollongong Hospital Illawarra Shoalhaven Local Health District Wollongong New South Wales Australia

**Keywords:** fasting glucose, GDM, gestational diabetes, oral glucose tolerance test, pregnancy, pregnancy outcomes

## Abstract

**Background:**

During COVID‐19, the diagnosis and treatment of GDM differed from conventional criteria. In Australia, during the alternative testing period, women with fasting glucose < 4.7 mmol/L were not diagnosed with GDM.

**Aim:**

To describe the maternal and neonatal outcomes of pregnant women with fasting blood glucose < 4.7 mmol/L for whom the diagnosis and treatment pathways differed before and during COVID‐19.

**Materials and Methods:**

An Australian population‐based data linkage study involving 3891 women with fasting blood glucose < 4.7 mmol/L between 24 and 32 weeks of gestation categorised into three groups: women diagnosed with GDM by postprandial hyperglycaemia (PPGDM; *n* = 226); normal glucose tolerance group (NGT; *n* = 3125) and women not tested for postprandial hyperglycaemia, mostly during COVID‐19 (LFBG; *n* = 540). Perinatal outcomes were compared using generalised linear models.

**Results:**

There were no differences between PPGDM and NGT groups in the risk of large for gestational age infants (RR 0.98, 95% CI: 0.63–1.52) although the mean birth weight (MD −103.43, 95% CI: −175.46 to −31.40)) was lower in the PPGDM group. The maternal and neonatal outcomes in the LFBG group were mostly comparable to the NGT group.

**Conclusion:**

In our study, the Australian COVID‐19 GDM screening protocol, which includes initial fasting glucose testing, reduced the need for an OGTT in 67% of pregnant women. Diagnosis and treatment for postprandial hyperglycaemia in women with lower FBG should consider the benefits, as well as the financial, logistical and psychological costs involved.

## 1 Introduction

Gestational Diabetes Mellitus (GDM) is a common complication in pregnancy [1, 2]. In Australia, the prevalence of GDM has risen to 17.9% during the recent coronavirus (COVID‐19) pandemic (2020–2021) and to 19.3% since (2021–2022) [3]. GDM is defined as hyperglycaemia with onset or first detected in pregnancy and is characterised by an increased risk of adverse perinatal outcomes [2]. In the long term, GDM is associated with an increased risk of maternal Type 2 diabetes, cardiovascular disease, and abnormal glucose metabolism of offspring in childhood [4]. The conventional criteria for diagnosing GDM between 24 and 28 weeks is by 75 g oral glucose tolerance test (OGTT) [5] as per the Australasian Diabetes in Pregnancy Society (ADIPS) and World Health Organisation guidelines.

**FIGURE 1:**
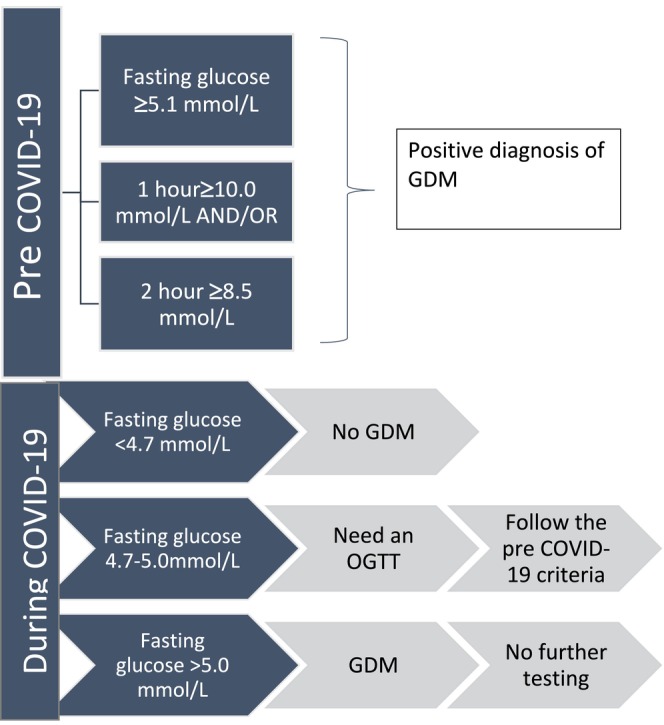
Difference in GDM diagnostic pathways before and during COVID‐19 in Australia endorsed by Australasian Diabetes in Pregnancy Society.

The recent COVID‐19 pandemic resulted in major changes to the health care service delivery [6]. In Australia, the ADIPS temporarily changed the routine OGTT procedures in April 2020 to minimise the potential exposure to COVID‐19 [7]. The temporary criteria for diagnosing GDM during COVID‐19 was using a two‐step approach with an initial fasting blood glucose. If the initial fasting blood glucose was < 4.7 mmol/L, it was considered a normal result with no further testing or diagnosis of GDM [8]. Please see Figure 1 for more details. The above criteria were based on retrospective data analysis of OGTT data which estimated that only 6% of women will miss a diagnosis of GDM using this approach [9]. There is a unclear evidence around whether the diagnosis and treatment of postprandial hyperglycaemia in women with lower fasting blood glucose improve perinatal outcomes [8, 10]. Analysis of the temporary COVID‐19 GDM diagnostic criteria is opportunistic to examine the conventional GDM diagnostic methods to study the effects of undiagnosed and untreated postprandial hyperglycaemia in women with lower fasting glucose.

The aim of the current study is to explore the effects of postprandial hyperglycaemia screening by OGTT and GDM treatment in women with lower fasting glucose (< 4.7 mmol/L) on maternal and neonatal outcomes.

## 2 Materials and Methods

This population‐based study involved pregnant women who delivered at an Australian tertiary hospital between 2017 and 2021. The study utilised linked data from three hospital administrative datasets: the Wollongong hospital maternity data, the Illawarra Shoalhaven Diabetes Service data, and the perinatal NSW (New South Wales) dataset. The Illawarra Shoalhaven Diabetes Service collects data on diabetes treatments (diet, insulin) in women who had GDM. The data linkage was achieved through deterministic record linking of medical record numbers (MRN). The MRNs were replaced with an encrypted unique key which could not be linked to the original MRN by the Centre for Health Research in the Illawarra and Shoalhaven Population (CHRISP) who did the data linkage and de‐identification. Ethics approval for the project was obtained through the University of Wollongong Human Research Ethics Committee (ETH 00715/2021). As previously mentioned, to limit the COVID‐19 exposure for pregnant women, the local health district adopted the revised criteria in April 2020 and reverted to pre‐pandemic criteria in February 2021. We have reported the findings according to the STROBE (Strengthening the Reporting of Observational Studies in Epidemiology) Statement.

### 2.1 Study Population

**FIGURE 2:**
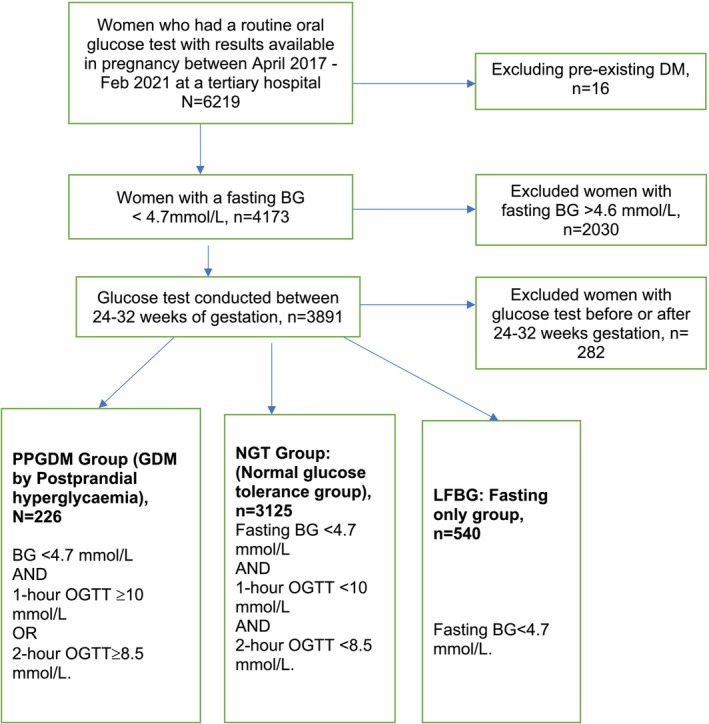
STROBE flowchart of methodology.

The study population was identified as women who delivered at the Wollongong hospital, based on the availability of women's OGTT or fasting glucose test results recorded on the maternity database, conducted routinely in pregnancy between April 2017 and Feb 2020 which identified a total of 6219 women. Out of these, 4173 women (67.2%) had a fasting blood glucose < 4.7 mmol/L and 3891 women had their glucose test during 24–32 weeks of gestation and were included in the study. Please see the flowchart (Figure 2) for further details.

### 2.2 Exposures

Data were categorised into three groups as follows: Postprandial GDM (PPGDM) group: Women diagnosed with GDM by postprandial hyperglycaemia through OGTT: fasting blood glucose (FBG) < 4.7 mmol/L, 1‐h ≥ 10 mmol/L or 2‐h ≥ 8.5 mmol/L), Normal Glucose Tolerance (NGT) Group: Fasting BG < 4.7 mmol/L, 1 h < 10 mmol/L and 2 h < 8.5 mmol/L and Low fasting blood glucose (LFBG) Group: women who had a FBG < 4.7 mmol/L, but no OGTT during COVID‐19.

Women in the PPGDM group underwent routine diabetes education, received dietary counselling, and self‐monitored their blood glucose. If the women had three blood glucose readings in a week that were above the target range of fasting > 5.2 mmol/L or one‐hour post meal > 7.4 mmol/L they were reviewed by the endocrinologist to assess the need for insulin therapy.

### 2.3 Outcomes

The primary outcome of interest was the birth of an infant who was large for gestational age (LGA), defined as birth weight > 90th percentile for gestational age [11]. Maternal outcomes included mode of delivery, gestational hypertension, preeclampsia, polyhydramnios, oligohydramnios, and maternal length of stay in hospital. Other neonatal outcomes included sex‐specific birth weight for gestational age z‐scores, macrosomia (> 4 kg at birth), small for gestational age (SGA)—birth weight < 10th percentile for gestational age, birth injury, congenital malformations, neonatal hypoglycaemia (blood glucose < 2.6 mmol/L), respiratory distress (including respiratory distress syndrome, transient tachypnoea of new born and apnoea), hyperbilirubinemia, use of birth manoeuvres for shoulder dystocia and admission to special care nursery. Adverse birth outcomes of neonatal death and stillbirth were excluded due to nil/small numbers in line with Australian Bureau of Statistics‐confidentiality rules. Estimation of LGA and SGA as well as age‐ and sex‐specific *z*‐scores were calculated using the AIHW data for live singleton babies born between 2004 and 2013 [9].

### 2.4 Statistics

Statistical analyses were conducted using Stata SE version 18 licenced by Stata Corp. Descriptive statistics are used to summarise demographic variables. We used means and standard deviations (SD) for reporting and describing continuous variables and medians and interquartile ranges (IQR) for skewed data description. Categorical variables are presented as total number (*n*) and percentages. Generalised linear regression models are used to compare the groups and are presented as unadjusted and adjusted risk ratios or mean differences along with 95% confidence intervals. We used a gaussian or Poisson link function for continuous variables, and binomial link function for binary outcomes. We have adjusted for confounding variables such as maternal pre‐pregnancy BMI, maternal age, parity, history of polycystic ovarian syndrome, family history of diabetes, previous history of GDM, Aboriginal and Torres Strait Islander ethnicity and smoking during the first half of pregnancy, considering the causal structure around the exposure and outcome. Adjusted and unadjusted results of analysis are shown in Table 2.

We conducted three separate analyses. The first analysis between the PPGDM group and NGT group compared the differences in pregnancy outcomes in the treated group to the background population with normal postprandial glucose. The second analysis between the treated PPGDM group to the LFBG group described the effects of diagnosing and treating postprandial hyperglycaemia in women with low fasting glucose compared to population who did not undergo further testing/diagnosis. The third analysis assessed whether the perinatal outcomes of LFBG group were comparable to the background population with NGT.

## 3 Results

### 3.1 Descriptive Characteristics

Out of 3891 women, 226 women (5.8%) were in the PPGDM group, 3125 (80.3%) women were in the NGT group, and 540 (13.8%) women were in the LFBG group. Diagnostic results (fasting and OGTT) and clinical observations are shown in Table 1. Women in the PPGDM group were older and had more risk factors compared to the other groups. Only 19 out of 226 (8.4%) women in the PPGDM group required insulin in pregnancy.

**TABLE 1:** Demographic and descriptive characteristics of the study groups.

	PPGDM/Postprandial GDM group, *n* = 226	NGT/Normal Glucose tolerance group, *n* = 3125	LFBG/Low fasting blood glucose group, *n* = 540
Maternal age (in years)	31.5 ± 4.9	29.4 ± 5.1	28.7 ± 5.2
Gravida: Median (IQR)	2 (1;3)	2 (1;3)	2 (1;3)
Primigravida	64 (28.3%)	973 (31.1%)	195 (36.1%)
Parity: Median (IQR)	1 (0; 2)	1 (0; 1)	0 (0;1)
Body Mass Index (kg/m^2^)	25.4 ± 5.6	24.7 ± 5.3	24.8 ± 5.4
Country of birth—Australia	159 (70.35%)	2586 (82.75%)	478 (88.5%)
Aboriginal or Torres Strait Islander status	7 (3.1%)	121 (3.8%)	10 (1.8%)
Other most common country of birth	Philippines (3.5%) Vietnam (3.1%)	UK (1.6%) New Zealand (1.2%)	UK (1.5%) China (0.9%)
Positive family history of diabetes	82 (36.4%)	817 (26.2%)	120 (22.3%)
Previous history of GDM	38 (16.8%)	160 (5.1%)	22 (4.0%)
History of polycystic ovarian syndrome	22(9.7%)	163 (5.2%)	25 (4.6%)
History of chronic/essential hypertension	7 (3.1%)	10 (0.3%)	3 (0.5%)
Smoking in first half of pregnancy:	16 (7.0%)	253 (8.1%)	37 (6.8%)
Fasting glucose (mmol/L)	4.3 ± 0.23	4.2 ± 0.27	4.2 ± 0.26
1 h plasma glucose(mmol/L)	10.0 ± 1.3	6.6 ± 1.4	n/a
2‐h plasma glucose (mmol/L)	8.4 ± 1.6	5.6 ± 1.2	n/a
Weeks of gestation at diagnosis	27.9 ± 1.4	27.8 ± 1.3	27.3 ± 1.3

*Note:* Description of groups: All women have a fasting glucose < 4.7 mmol/L, PPGDM: Women diagnosed with gestational diabetes by post prandial hyperglycaemia, NGT: Women with normal glucose tolerance, LFBG: Women with only a fasting blood glucose in pregnancy.

Abbreviations: IQR: interquartile range, GDM: Gestational Diabetes Mellitus.

### 3.2 Maternal and Neonatal Outcomes

#### 3.2.1 Comparison Between PPGDM Group and NGT Group

The primary outcome of LGA risk was similar (RR: 0.98, 95% CI: 0.63–1.52) between the two groups. Though the PPGDM group had lower mean birth weight (MD −103.4, 95% CI: −175.4 to −31.4) compared to the NGT group, there was a 2.6‐fold higher risk of neonatal hypoglycaemia (RR: 2.63, 95% CI: 1.64–4.22), 62% higher risk of shoulder dystocia, (RR: 1.62, 95% CI: 1.04–2.52) and a 41% higher rate of nursery admission after birth (RR: 1.41, 95% CI: 1.04–1.90) compared to the NGT group.

#### 3.2.2 Comparison Between PPGDM Group and LFBG Group

The risk of LGA was similar for the PPGDM and LFBG groups (RR: 1.02, 95% CI: 0.59–1.75) despite the lower mean birth weight in the PPGDM group (MD: −121.1, 95% CI: −206.6 to −35.7). Babies born to women in the PPGDM group also had a three‐fold higher risk of neonatal hypoglycaemia (RR: 3.40, 95% CI: 1.66 to 6.95) compared to the LFBG group. Women diagnosed and treated for PPGDM had a 60% higher risk for elective caesarean section (RR: 1.60, 95% CI: 1.22 to 2.09) compared to the LFBG group. The PPGDM group also had a higher percentage of babies (11.9%) who were SGA compared to the LFBG group (7.6%) with RR: 1.52 (95% CI: 0.93–2.47).

#### 3.2.3 Comparison Between LFBG Group and NGT Group

Across most of the maternal and neonatal measures, risk of adverse outcomes were similar between LFBG and NGT groups (Table 2). However, the LFBG group had a 17% reduction in caesarean section (RR: 0.83, 95% CI: 0.71–0.97), especially elective caesarean section (RR: 0.81, 95% CI: 0.68–0.97) after adjusting for confounding factors.

**TABLE 2:** Comparison of maternal and neonatal outcomes.

	PPGDM group (*n* = 226) mean (SD) or *n* (%)	NGT group (*n* = 3125) mean (SD) Or *n* (%)	LFBG group, (*n* = 540) mean (SD) Or *n* (%)	PPGDM vs. NGT unadjusted (U)/Adjusted (A) relative risk/mean difference^a^ (95% confidence interval)	PPGDM vs. LFBG unadjusted (U)/adjusted (A) relative risk/mean difference^a^ (95% confidence interval)	LFBG vs. NGT unadjusted (U)/adjusted (A) relative risk/mean difference^a^ (95% confidence interval)
Maternal length of stay (In days)	2.2 (1.3)	1.9 (1.4)	2.0 (1.2)	U: 0.26 (0.07–0.44) A: 0.23 (0.04–0.42)	U: 0.15 (−0.04 to 0.34) A: 0.15 (−0.05 to 0.35)	U: 0.11 (−0.01 to 0.23) A: 0.08 (−0.03 to 0.20)
Gestational hypertension	7 (3.1%)	57 (1.8%)	14 (2.6%)	U: 1.69 (0.78–3.67) A: 1.54 (0.70–3.36)	U: 1.19 (0.48–2.92) A: 0.78 (0.28–2.18)	U: 1.42 (0.79–2.53) A: 1.35 (0.76–2.39)
Oligohydramnios	4 (1.7%)	91 (2.9%)	12 (2.2%)	U: 0.60 (0.22–1.63) A: 0.64 (0.24–1.70)	U: 0.79 (0.25–2.44) A: 0.83 (0.28–2.44)	U: 0.76 (0.42–1.38) A: 0.73 (0.40–1.33)
Polyhydramnios	2 (0.8%)	36 (1.1%)	10 (1.8%)	U: 0.76 (0.18–3.17) A: 0.65 (0.16–2.65)	U: 0.47 (0.10–2.16) A: 0.35 (0.07–1.75)	U: 1.60 (0.80–3.21) A: 1.71 (0.84–3.48)
Spontaneous vaginal birth	117 (51.7%)	1797 (57.5%)	316 (58.5%)	U: 0.90 (0.79–1.02) A: 0.92 (0.81–1.05)	U: 0.88 (0.76–1.02) A: 0.88 (0.76–1.02)	U: 1.01 (0.94–1.09) A: 1.03 (0.96–1.11)
Instrumental birth	24 (10.6%)	362 (11.6%)	84 (15.6%)	U: 0.92 (0.62–1.35) A: 0.93 (0.63–1.36)	U: 0.68 (0.44–1.04) A: 0.76 (0.50–1.15)	U: 1.34 (1.07–1.67) A: 1.21 (0.98–1.50)
Caesarean Section	85 (37.6%)	966 (30.9%)	140 (25.9%)	U: 1.21 (1.02–1.45) A: 1.11 (0.93–1.33)	U: 1.45 (1.16–1.80) A: 1.37 (1.08–1.74)	U: 0.83 (0.72–0.97) A: 0.83 (0.71–0.97)
Emergency caesarean section	11 (13.1%)	216 22.5%)	36 (25.7%)	U: 0.58 (0.33–1.02) A: 0.72 (0.39–1.30)	U: 0.50 (0.27–0.94) A: 0.74 (0.36–1.51)	U: 1.14 (0.84–1.54) A: 0.92 (0.65–1.29)
Elective caesarean section	73 (86.9%)	742 (77.4%)	104 (74.3%)	U: 1.36 (1.11–1.65) A: 1.22 (1.00–1.49)	U: 1.67 (1.29–2.16) A: 1.60 (1.22–2.09)	U: 0.81 (0.67–0.97) A: 0.81 (0.68–0.97)
Birth weight (grams)	3273 (513.0)	3377 (530.5)	3389 (518.0)	U: −104.1 (−175.5 to −32.6) A: −103.4 (−175.4 to −31.4)	U: 115.8 (−196.0 to −35.6) A: −121.1 (−206.6 to −35.7)	U: 11.7 (−36.5–60.0) A: 11.9 (−36.1 to 60.0)
Birthweight *z*‐score	−0.07 (0.9)	0.01 (0.9)	0.04 (0.9)	U: −0.08 (−0.21 to −0.04) A: −0.10 (−0.23 to 0.02)	U: −0.11 (−0.26 to 0.02) A: −0.14 (−0.30 to 0.00)	U: −0.03(−0.05 to 0.11) A: 0.04 (−0.04 to 0.12)
Macrosomia	15 (6.6%)	32 (10.4%)	55 (10.2%)	U: 0.63 (0.38–1.04) A: 0.62 (0.38–1.04)	U: 0.66 (0.37–1.12) A: 0.57 (0.32–1.03)	U: 0.94 (0.74–1.27) A: 0.97 (0.74–1.27)
Large for gestational age	21 (9.3%)	279 (8.9%)	46 (8.5%)	U: 1.04 (0.68–1.58) A: 0.98 (0.63–1.52)	U: 1.09 (0.66–1.78) A: 1.02 (0.59–1.75)	U: 0.95 (0.70–1.28) A: 0.96 (0.71–1.30)
Small for gestational age	27(11.9%)	282 (9.0%)	41 (7.6%)	U: 1.32 (0.91–1.91) A: 1.31 (0.90–1.91)	U: 1.57 (0.99–2.49) A: 1.52 (0.93–2.47)	U: 0.84 (0.61–1.15) A: 0.84 (0.61–1.15)
Birthweight < 2500 (g)	15 (6.6%)	182 (5.8%)	29 (5.4%)	U: 1.13 (0.68–1.89) A: 1.08 (0.64–1.81)	U: 1.23 (0.67–2.26) A: 1.37 (0.75–2.51)	U: 0.92 (0.62–1.34) A: 0.92 (0.63–1.35)
Length (cm)	49.7(2.7)	50.2 (2.5)	50.2 (2.4)	U: −0.49 (−0.84 to −0.14) A: −0.47(−0.83 to −0.12)	U: −0.54 (−0.94 to −0.14) A: −0.55 (−0.97 to −0.12)	U: 0.05 (−0.18 to 0.28) A: 0.04 (−0.19 to 0.27)
Head circumference (cm)	34.0 (1.6)	34.2 (1.6)	34.3 (1.6)	U: −0.18 (−0.40 to 0.02) A: −0.22 (−0.44 to −0.01)	U: −0.23 (−0.48 to 0.01) A: −0.25 (−0.52 to 0.00)	U: 0.04 (−0.10 to 0.18) A: 0.04 (−0.09 to 0.19)
Gestational age (weeks)	38.6 (1.5)	39.0 (1.5)	39.0 (1.5)	U: −0.44 (−0.65 to −0.23) A: −0.40 (−0.61 to −0.19)	U: −0.44 (−0.68 to −0.20) A: −0.42 (−0.67 to −0.17)	U: −0.00 (−0.14 to 0.13) A: −0.02 (−0.16 to 0.11)
Preterm birth (< 37 Weeks)	21 (9.3%)	220 (7.0%)	40 (7.4%)	U: 1.31 (0.86–2.04) A: 1.29 (0.84–2.00)	U: 1.25 (0.75–2.07) A: 1.23 (0.72–2.09)	U: 1.05 (0.76–1.45) A: 1.05 (0.76–1.46)
Birth trauma	4 (1.8%)	69 (2.2%)	19 (3.5%)	U: 0.80 (0.29–2.17) A: 1.09 (0.44–2.71)	U: 0.49 (0.17–1.46) A: 0.50 (0.16–1.56)	U: 1.59 (0.96–2.62) A: 1.52 (0.92–2.51)
Shoulder dystocia	20 (8.9%)	176 (5.6%)	35 (6.5%)	U: 1.57 (1.00–2.44) A: 1.62 (1.04–2.52)	U: 1.36 (0.80–2.31) A: 1.40 (0.79–2.48)	U: 1.15 (0.81–1.63) A: 1.12(0.78–1.59)
Congenital anomalies	7 (3.1%)	70 (2.2%)	14 (2.6%)	U: 1.38 (0.64–2.97) A: 1.33 (0.61–2.90)	U: 1.19 (0.48–2.92) A: 1.06 (0.40–2.81)	U: 1.15 (0.65–2.03) A: 1.13 (0.64–1.99)
Respiratory distress at birth	24 (10.6%)	255 (8.2%)	51 (9.4%)	U: 1.30 (0.87–1.93) A: 1.26 (0.85–1.87)	U: 1.12 (0.71–1.78) A: 1.18 (0.66–1.77)	U: 1.15 (0.86–1.54) A: 1.13 (0.85–1.51)
Respiratory distress in postnatal period	27 (12.0%)	289 (9.2%)	66 (12.2%)	U: 1.29 (0.89–1.87) A: 1.27 (0.87–1.84)	U: 0.97 (0.64–1.48) A: 0.92 (0.59–1.44)	U: 1.32 (1.02–1.69) A: 1.27 (0.99–1.64)
Neonatal hypoglycaemia	21 (9.2%)	93 (2.9%)	13 (2.4%)	U: 3.12 (1.98–4.91) A: 2.63 (1.64–4.22)	U: 3.85 (1.96–7.57) A: 3.40 (1.66–6.95)	U: 0.80 (0.45–1.43) A: 0.82 (0.46–1.46)
Hyperbilirubinemia in the newborn	14 (6.2%)	135 (4.3%)	31 (5.7%)	U: 1.43 (0.84–2.44) A: 1.48 (0.86–2.55)	U: 1.07 (0.58–1.98) A: 1.21 (0.62–2.33)	U: 1.32 (0.90–1.94) A: 1.24 (0.85–1.81)
Admission to special care nursery	40 (17.7%)	364 (11.6%)	62 (11.5%)	U: 1.51 (1.12–2.04) A: 1.41 (1.04–1.90)	U: 1.54 (1.06–2.22) A: 1.42 (0.96–2.11)	U: 0.98 (0.76–1.26) A: 0.97 (0.75–1.25)

*Note:* Description of groups: All women have a fasting glucose < 4.7 mmol/L. PPGDM: Women diagnosed with gestational diabetes by post prandial hyperglycaemia. NGT: Women with normal glucose tolerance. LFBG: Women with only a fasting blood glucose in pregnancy. Mean Difference (MD) for continuous outcomes and Relative risk (RR) for binary outcomes. Adjusted for Maternal age, maternal pre‐pregnancy body mass index/BMI, Aboriginal or Torres Strait Islander status, family history of diabetes, history of polycystic ovarian syndrome, previous history of GDM and parity.

^a^ Relative risk (RR) or mean difference (MD) with 95% confidence interval.

## 4 Discussion

### 4.1 Main Findings

Our analysis revealed that GDM screening with an initial fasting blood glucose reduced the need for OGTT in 67% of pregnant women in our study. Women with fasting blood glucose < 4.7 mmol/L who did not undergo further testing or treatment had lower risk for adverse maternal and neonatal outcomes similar to women with normal glucose tolerance. This study also demonstrated the risk of LGA was similar across all groups despite the difference in postprandial hyperglycaemia screening and treatment.

Although the PPGDM group had a higher risk profile with higher maternal age, pre pregnancy BMI, family history of diabetes, previous history of GDM, the treatment of postprandial hyperglycaemia in this group alone did not effectively mitigate the specific risk of LGA or shoulder dystocia despite a reduction in mean birth weight and macrosomia. This finding is in agreement with Crowther et al. (2022) [11] that regardless of diagnostic criteria used for diagnosing GDM, the risk of LGA infant remained the same. It is well‐known that the treatment of GDM reduces the birth weight of infant [12], however, the lower rate of macrosomia in the PPGDM group could have been influenced by local practices of additional foetal growth monitoring and induction of labour. While the higher incidence of nursery admission, neonatal hypoglycaemia and elective caesarean section in the PPGDM group could also be due to the local policies for additional monitoring and perceived risk of complications in women with GDM, the staggering three‐fold higher risk of neonatal hypoglycaemia in the PPGDM group compared to LFBG group is worth further research due to its long‐term neurodevelopmental consequences in children [13]. Previous study has reported that postprandial hyperglycaemia was associated with preterm delivery [14], gestational hypertension, and hyperbilirubinemia [15], however our analysis revealed an increase in shoulder dystocia and trend towards increased preterm birth and small for gestational age babies in in the PPGDM group despite treatment.

Women who only had a fasting blood glucose test had mostly similar maternal and neonatal outcomes to that of women with normal glucose tolerance. McIntyre et al. [16] through analysis of the HAPO study data and study by Meloncilli et al. [17] also reported similar findings between these two groups for pregnancy related complications. Rodrigo et al. [18] observed similar trends that women with fasting blood glucose < 4.7 mmol/L but elevated 1‐ or 2‐h levels on an OGTT can be considered a low‐risk group. There was an increased trend towards emergency caesarean section and instrumental delivery in the LFBG group compared to the NGT group. We found a high number of emergency caesarean sections during COVID‐19 across all groups possibly due to the use of telehealth, reduced face to face clinic visits and lockdowns during COVID 19.

The Australian COVID‐19 screening process for GDM with an initial fasting glucose reduced the need for further OGTT in 67% of pregnant women in our study. Similar study conducted in Victoria reported 80% reduction in the need for OGTT using the COVIID‐19 GDM criteria [19]. Despite the OGTT being considered as the gold standard for diagnosing GDM, it is not favoured or tolerated by many as it is time consuming, and uncomfortable [20]. Furthermore, some clinicians believe that OGTT results are not reproducible, or corrected for body weight; and the predictive value changes with ethnicity [21]. Beunen et al. [22] has stated that a fasting blood glucose cut‐off of 4.6 mmol/L will only miss a GDM diagnosis on 6.7% of total GDM cases and has 94.3% negative predictive value. While screening for GDM with an initial fasting blood glucose is appealing to women, cheap and reproducible without the discomfort of OGTT, women with higher fasting glucose may need a second appointment and OGTT. In addition, women from Asian backgrounds tend to have lower fasting blood glucose compared to Caucasian women and an increased risk of postprandial hyperglycaemia [22], thus still may need a full OGTT. Therefore, the benefits of conducting OGTT and subsequent treatment of postprandial hyperglycaemia in women with lower fasting glucose must be weighed against the benefits as well as the financial, logistical and psychological costs of the OGTT to the wider group of pregnant population.

The strength of this descriptive study is that this is a data linkage study between three datasets which provided complete and complimentary information across three platforms. Although this is a single centre study, where data were collected prospectively, the study population is demographically on par with the wider Australian population and thus adds to generalisability of findings [23]. Extensive data wrangling and cross checking were performed to limit information bias. The study unlikely has selection bias as the data were collected from the entire population of pregnant women. We did not have data on the ethnic background of women in the study, rate of induction of labour, gestational weight gain and impact of COVID‐19 all of which could affect the pregnancy outcomes.

While COVID‐19 brought on some unprecedented challenges, it provided an opportunity to review the conventional GDM diagnosis through an analysis of an alternate approach used in Australia. This study identified that the diagnosis and treatment of postprandial hyperglycaemia in women with low fasting glucose did not reduce the risk of LGA despite a lower birth weight but required additional monitoring and interventions thus highlights the need for evaluating the conventional method to GDM diagnosis and treatment. A simplified approach to GDM diagnosis with an initial fasting blood glucose will reduce the need for OGTT in a vast group of pregnant women and enables risk categorization. Further studies are needed to explore whether an earlier diagnosis may benefit women with high risk factors who are at risk of LGA infants.

## Conflicts of Interest

The authors declare no conflicts of interest.
